# Spontaneous white matter damage, cognitive decline and neuroinflammation in middle-aged hypertensive rats: an animal model of early-stage cerebral small vessel disease

**DOI:** 10.1186/s40478-014-0169-8

**Published:** 2014-12-18

**Authors:** Daniel Kaiser, Gesa Weise, Karoline Möller, Johanna Scheibe, Claudia Pösel, Sebastian Baasch, Matthias Gawlitza, Donald Lobsien, Kai Diederich, Jens Minnerup, Alexander Kranz, Johannes Boltze, Daniel-Christoph Wagner

**Affiliations:** Fraunhofer Institute for Cell Therapy and Immunology, Perlickstraße 1, 04103 Leipzig, Germany; Department of Neurology, University of Leipzig, Leipzig, Germany; Department of Radiology, University of Leipzig, Leipzig, Germany; Department of Neuroradiology, University of Leipzig, Leipzig, Germany; Department of Neurology, University of Münster, Münster, Germany; Translational Centre for Regenerative Medicine, Leipzig, Germany

**Keywords:** Cerebral small vessel disease, White matter disease, Spontaneously hypertensive rat, Neuroinflammation, T Cells

## Abstract

**Introduction:**

Cerebral small vessel disease (cSVD) is one of the most prevalent neurological disorders. The progressive remodeling of brain microvessels due to arterial hypertension or other vascular risk factors causes subtle, but constant cognitive decline through to manifest dementia and substantially increases the risk for stroke. Preliminary evidence suggests the contribution of the immune system to disease initiation and progression, but a more detailed understanding is impaired by the unavailability of appropriate animal models. Here, we introduce the spontaneously hypertensive rat (SHR) as a model for early onset cSVD and unveiled substantial immune changes in conjunction with brain abnormalities that resemble clinical findings.

**Results:**

In contrast to age-matched normotensive Wistar Kyoto (WKY) rats, male SHR exhibited non-spatial memory deficits. Magnetic resonance imaging showed brain atrophy and a reduction of white matter volumes in SHR. Histological analyses confirmed white matter demyelination and unveiled a circumscribed blood brain barrier dysfunction in conjunction with micro- and macrogliosis in deep cortical regions. Flow cytometry and histological analyses further revealed substantial disparities in cerebral CD45high leukocyte counts and distribution patterns between SHR and WKY. SHR showed lower counts of T cells in the choroid plexus and meningeal spaces as well as decreased interleukin-10 levels in the cerebrospinal fluid. On the other hand, both T and NK cells were significantly augmented in the SHR brain microvasculature.

**Conclusions:**

Our results indicate that SHR share behavioral and neuropathological characteristics with human cSVD patients and further undergird the relevance of immune responses for the initiation and progression of cSVD.

**Electronic supplementary material:**

The online version of this article (doi:10.1186/s40478-014-0169-8) contains supplementary material, which is available to authorized users.

## Introduction

Cerebral small vessel disease (cSVD) has rapidly gained attention as a growing medical and socioeconomic burden. It is supposed to cause about one fifth of strokes worldwide [1] and more than doubles the risk for a recurrent attack [2]. Furthermore, progressive white matter damage relates to substantial cognitive decline, thus being held responsible for almost half of dementias among the elderly population [3].

Considering its enormous impact, surprisingly little is known about the pathogenesis of cSVD. Low mortality certainly contributes to this lack of knowledge as post mortem studies in patients reveal late-stage tissue alterations rather than incipient steps of the disease cascade [4]. Neuroimaging is currently the gold standard to assess cSVD, but only captures tissue changes secondary or even tertiary to the underlying pathology. Consequently, there is a demand for animal models that allow systematic investigation of the cellular and molecular basis of cSVD, including the possibility to carry out preclinical proof-of-concept trials.

Various relevant animal models of cSVD are described in the literature, but it seems that they separately mimic different aspects of human cSVD such as lacunar infarcts, white matter damage or vessel dysfunction without covering the entire pathophysiological cascade. Hereof, stroke prone spontaneously hypertensive rats (SHR-SP) feature most of the cardinal histopathological signs of cSVD [5,6] likely as a consequence of chronically increased arterial blood pressure (BP) that causes vascular dysfunction on a rodent time scale [7]. However, the SHR-SP model is biased towards the bleeding facet of cSVD [8] which might be due to genetically fixed alterations of the endothelial tight junctions, being already evident in the pre-hypertensive age of 5 weeks or less [9].

In human cSVD, bleedings and lacunar infarcts typically occur in the basal ganglia while white matter hyperintensities preferentially develop in the centrum semiovale. Anatomical factors might explain these differing predilection sites: arterioles entering the deep white matter from the superficial cortex are coated by a single leptomeningeal layer rendering them more susceptible to hypertension-related vascular damage [4,10]. A recent cross-sectional imaging study revealed that increased systolic BP progressively disrupts white matter integrity already in young adults [11]. A similar relation, however, has not yet been described in animal models.

Several lines of evidence indicate that the immune system significantly contributes to the development and progression of cSVD. Serum levels of soluble adhesion molecules were increased in patients with white matter lesions [12] and blood monocytosis correlated with the incidence of lacunar infarcts [13]. In 2005, a large population-based cohort study unveiled that c-reactive protein (CRP) levels correlate with the existence and progression of white matter damage [14]. The association of inflammation and cSVD is not surprising since chronic inflammation also plays an important role in the pathophysiology of its primary risk factor hypertension [15]. However, whether such inflammatory processes primarily initiate vascular remodeling, secondarily promote its propagation, or simply constitute a response to ongoing reorganization remains unclear.

In this study, we investigated whether hypertension in rodents triggers early changes of central brain regions that resemble findings obtained from cSVD patients. For this purpose, we took advantage of spontaneously hypertensive rats (SHR) that, akin to their stroke-prone relatives, develop arterial hypertension prior to the age of 10 weeks, but have no disposition for spontaneous cerebral hemorrhages. We found strong evidence that SHR develop circumscribed blood brain barrier (BBB) dysfunction, white matter damage and microglial activation in their first third of life. Interestingly, we also found substantial differences in the amount and distribution of blood-born leukocytes in brains of SHR compared to age-matched normotensive controls. Thus, our results not only suggest a relevance of SHR as a model for early-stage hypertensive cSVD, but also indicate neuroinflammatory contributions to the development of cSVD.

## Material and methods

### Animals

Animal experiments were approved by the local animal welfare authority (Landesdirektion Sachsen, license number TVV 06/13) and conducted according to the Guide for the Care and Use of Laboratory Animals published by the US National Institutes of Health (NIH Publication No. 85–23, revised 1996). A total of 16 male SHR and 16 Wistar Kyoto rats (WKY, both Charles River, Sulzfeld, Germany) was used in this study and randomly assigned to four experimental groups (Additional file 1: Figure S1). After delivery, 11-week old animals were housed under standardized conditions with free access to water and food. Systolic BP was measured biweekly from week 12 to 22 by indirect tail-cuff method (ML125 NIBP, ADInstruments, Oxford, UK). Pressure and pulse rate signals were continuously recorded and digitalized using PowerLab and LabChart software (both ADInstruments). Systolic BP was determined as the mean of three cuff inflation measurements.

### Blood brain barrier integrity and blood vessel volume

FITC-lectin (1.6 mg/kg, Sigma, Taufkirchen, Germany) and Evans Blue (EB, 2% in saline, 4 mL/kg, Sigma) were injected into the tail vein of 24-week old rats (n = 3/3) under inhalation anesthesia with isoflurane. Subsequently, animals were sacrificed by an over-dose of ketamine and transcardially perfused with 200 mL of phosphate buffered solution (PBS). Brains were removed and shock-frozen in isopentane at −65°C. Coronal brain sections of 30 μm (anterior/posterior 1.32 to −5.04 mm relative to bregma) were cut, mounted and fixated with 4% formalin solution for 15 min. Confocal stacks of six randomly selected sections per animal were acquired using a LSM 710 confocal laser scanning microscope (Laser: Diode 405, Argon 488, HeNe 543; Objective: Plan-Apochromat 63x/1.40 oil, Zeiss, Jena, Germany). Red channel stacks (EB) were transformed into maximum intensity projection images using the FIJI image processing package (www.fiji.sc). The EB positive area per ROI was determined for the whole hemisphere and for the deep cortical region (DCR) adjacent to the corpus callosum (CC). FITC-lectin stained vessels were acquired in three ROIs (1536×1536×12 μm) randomly placed among the DCR, and the total vessel volume was assessed using the surface algorithm (absolute intensity threshold: 20–255; minimal volume: >100 voxels) of Imaris (Bitplane, Zurich, Switzerland).

### Behavioral tests

The novel object recognition test (NORT) was realized in a noise and light-shielded acryl box (480×480 mm), illuminated with 120 lux as described previously [16]. Briefly, 30-week old rats (n = 10/10) were allowed to habituate to the arena and to the to-be-familiarized object (cube) for 10 min on two consecutive days. Four hours after the second habituation trial, rats were placed into the arena, equipped with the familiar and the novel object (cup) for 5 min. The discriminatory index was calculated as following: interaction time with novel object divided by total interaction time with both objects. Morris water maze (MWM) was performed as reported earlier [17]. Briefly, a circular pool (diameter 180 cm, height 42 cm) was filled with water (24 ± 2°C) to a height of 32 cm. The pool was then divided into quadrants which were individually branded by optical cues. A transparent escape platform (diameter 10 cm) was placed 2 cm beneath the water surface in the center of one quadrant. Starting points were alternately set into the other quadrants. After one day of habituation, maze performance of 34-week old rats (n = 9/9) was investigated at three consecutive days. Each day, two trials of maximally 90 sec with an inter-trial delay of 30 sec were run. Whether successful or not, animals were allowed to rest on the platform for 30 sec. Video recorded experiments were analyzed using FIJI and MTrack2 (valelab.ucsf.edu) tracking plugin. The time needed (escape latency) and the distance covered to reach the platform was assessed.

### Magnetic resonance imaging

35-week old animals (n = 10/10) were anesthetized by intraperitoneal injection of ketamine hydrochloride (100 mg/kg, Merial, Hallbergmoos, Germany) and xylazine (10 mg/kg, Bayer, Leverkusen, Germany). Measurements were performed in a clinical 3.0 Tesla scanner (MAGNETOM Trio, Siemens, Erlangen, Germany) equipped with a small loop radiofrequency coil (3 T Loop 4 cm, Siemens). T2-weighted turbo spin echo sequences (T2-TSE) consisting of 36 coronal slices of the brain (matrix: 224 × 224; field of view: 77 mm; slice thickness: 1 mm) were registered. DICOM sequences were analyzed using FIJI. Total brain volume, CC volume and volume of the ventricular system were determined on binarized sequences obtained by thresholding.

### Sampling

35-week old SHR and WKY (n = 13/13) were sacrificed by an over-dose of ketamine. Randomly selected rats (n = 5/5) were placed in a stereotaxic frame, and the head was flexed downward at 45 degree. A midline scalp incision was made and the atlanto-occipital membrane was exposed. A 27 G needle was inserted into the cisterna magna for collection of 50–100 μL cerebrospinal fluid (CSF). CSF samples were centrifuged at 3000 rpm for 5 min and stored in aliquots at −80°C. Next, left cardiac ventricles were punctured via the opened thoracic cavity, and 5 mL of blood were collected for further analyses. The body was transcardially perfused with 200 mL of PBS. For flow cytometry of brain tissue (n = 3/3) removed brains were dissected and isolated hemispheres were stored in PBS at 4°C for 30 min. For gene expression analysis (n = 5/5), removed brains were shock-frozen in isopentane at −65°C and stored at −80°C. For histological analyses (n = 4/4) PBS perfusion was followed by perfusion of 200 mL of 4% formalin solution. Isolated brains were kept in 4% formalin solution for 24 h and vitrified in a 30% sucrose solution. Consecutive coronal sections (anterior/posterior 1.32 to −5.04 mm relative to bregma) were cut with a thickness of 10 or 25 μm, respectively, mounted on Superfrost Plus slides (Menzel, Braunschweig, Germany) and finally stored at −20°C. For analysis of neurogenesis, 40 μm thick free-floating sections (anterior/posterior 0.88 to 0.36 mm relative to bregma) were stored in tubes containing 25% glycerine, 25% ethylene glycol in 0.1 M phosphate buffer.

### Hematological parameters

Basic hematological parameters (Red blood cell count, RBC; hemoglobin, Hb; hematocrit, HCT; mean corpuscular hemoglobin, MCH; mean corpuscular hemoglobin concentration, MCHC; red blood cell distribution width, RDW; platelet count, PLT; mean platelet volume, MPV) were determined in blood samples anticoagulated with 2 mM EDTA using an animal blood counter (scil Vet abc, Vet scil animal care company, Viernheim, Germany).

### Flow cytometry

Multichannel flow cytometry was performed to sub-categorize leukocytes according to their antigen expression. 50 μL of EDTA-anticoagulated blood was diluted and pre-incubated with normal mouse serum. Multiple fluorescent-labeled monoclonal antibodies (anti-CD45 APC-Cy7-labeled, anti-CD45R FITC-labeled, anti-CD3 APC-labeled, anti-CD8 PerCP-labeled, anti-CD161a Biotin-labeled, secondly conjugated with streptavidin Horizon™V500 (BD Biosciences, Heidelberg, Germany), anti-CD11b PacificBlue-labeled (AbD Serotec, Puchheim, Germany) and anti-CD4 PE-Cy7-labeled (Biolegend, Fell, Germany) were added for 20 min at 4°C. Erythroid cells were lysed in distilled water. Cerebral leukocytes were quantified as described previously [18]. Briefly, brain hemispheres were manually chopped with a scalpel, and further dissociated by Collagenase I (Sigma) and DNAse I (Roche, Basel, Switzerland) in Hanks Balanced Salt Solution (HBSS). Percoll (GE Healthcare, Little Chalfont, UK) gradients were used for leukocyte separation. Cell viability was quantified using trypan blue exclusion. Samples of 1x10E5 cells were suspended in 100 μL of FACS buffer (3% fetal calf serum in PBS) and incubated for 20 min at 4°C with a mixture of monoclonal antibodies (anti-CD3 FITC-labeled (BD Biosciences), anti-RP1 PE-labeled, anti-CD161a Biotin-labeled, secondly conjugated with streptavidin Horizon™V500 (BD Biosciences), anti-CD45 PC5-labeled, anti-CD4 PC7-labeled (Biolegend), anti-CD8 APC-labeled, CD11b PacificBlue-labeled (AbD serotec)). Flow cytometry was performed using a 3-laser FACSCanto II (BD Biosciences) and analyzed by FlowJo software (Tree Star, Ashland, USA). CSF levels of IFNγ and IL-10 were analyzed by Cytometric Bead Array (BD Biosciences) as described in the manufacturer’s instructions.

### Gene expression analysis

For laser microdissection, shock-frozen brains were cut into 30 μm thick coronal sections, placed on membrane covered slides (MembraneSlide, Zeiss, Jena, Germany) and dried. A PALM MicroBeam laser dissection microscope (nitrogen laser, objective 20x, Zeiss) was used to isolate small vessels (diameter ≤ 50 μm) within the DCR that were visualized in the bright field mode on naive sections. The region of interest was manually encircled, laser-dissected and transferred into a tube (Adhesive Caps, Zeiss) by a second laser impulse. RNA of the acquired tissue sample (9x10E6 μm3 per animal) was purified using Rneasy Plus Micro kit (Qiagen, Hilden, Germany). For whole brain analyses, brain tissue was mechanically dissociated and total RNA of 100 mg tissue was extracted by homogenization in 1 mL Trizol using an Ultra-Turrax (Ika, Staufen, Germany) and further purified (RNeasy Mini Kit, Qiagen). RNA was transcripted into cDNA (Superscript III, Invitrogen, Darmstadt, Germany) according to the manufacturer’s instructions. mRNA expression was determined using an ABI 7900 real-time PCR system (Applied Biosystems, Darmstadt, Germany) at the following conditions: initial denaturation at 95°C for 10 min, followed by 50 cycles at 95°C for 15 sec and 55°C for 1 min. qRT-PCR reactions were conducted with QuantiTect SYBR Green PCR Kit and gene specific QuantiTect primers (Qiagen): ICAM-1, QT00174447; VCAM-1, QT00178500; nNOS, QT00186340; IL-1ß, QT00181657; TGFß, QT00187320; MMP-2, QT00996254; P-selectin, QT00180418. Data were analyzed using the relative standard curve method, normalized on the average cycle threshold of the housekeeping genes (YWHAZ, QT023821840; B2M QT00176295; RPL13a, QT00425873; RPL22, QT00385119) and relativized to the respective mean value of the WKY group.

### Histology

First, 10 μm sections were stained with hematoxylin/eosin (HE). A total of 50 vessels (diameter 10–50 μm) within the DCR were acquired. The luminal and total vessel area were manually encircled using an ECLIPSE Ti microscope (Nikon, Düsseldorf, Germany) and FIJI software. The vessel wall area was calculated by subtraction of the luminal area from the total area. The diameter (d) was assessed by circumference (C) d = C/π. To analyze the CC white matter density, luxol fast blue (LFB) staining and nuclear fast red counterstaining were performed. Whole CCs were acquired using an ECLIPSE Ti microscope and images were transformed into an 8 bit grey scale format (black/white 0/255). The myelin index (mean grey value) of the CC was automatically determined using FIJI. For immunohistochemistry, 25 μm sections were blocked with 5% goat serum, 0.3% Triton X-100 and PBS for 60 min, and incubated with either rabbit anti-Iba1 (Ionized calcium binding adaptor molecule 1; 1:200; Wako Chemicals, Neuss, Germany), biotinylated solanum tuberosum lectin (STL; 1:300; Linaris, Dossenheim, Germany), mouse anti-15-16A1 (1:500; Hycult Biotech, Beutelsbach, Germany; for T cells), mouse anti-GFAP (1:500, Sigma), mouse anti-CD161 (1:100, NovusBio, Cambridge, UK) for 24 h at 4°C. Sections were then incubated with AlexaFluor488- and 546-labeled species-specific secondary antibody (1:400, Invitrogen) for 2 h. STL was visualized by Cy5 Streptavidin (1:500, Jackson ImmunoResearch, Baltimore Pike, USA). Cell nuclei were stained with 4′, 6-diamidin-2-phenylindol (DAPI; Sigma) for 5 min. Confocal stacks of GFAP- and Iba1-stained sections were acquired in technical quadruplicates within the DCR using a LSM 710 (Zeiss). Contents of image stacks were converted into objects using the surface algorithm of Imaris (Bitplane). Total volume (for GFAP), cell-specific volume of dendrites and cells, as well as dendrite length (for Iba1) were measured using the Surface and Filaments algorithms (Bitplane). 15-16A1-positive T cells and Iba1-positive microglia were counted and T cells were further categorized (rod versus round; meninges, choroid plexus, parenchyma) using a Stereo Investigator system (MBF Bioscience, Williston, USA). Microglia distribution patterns were analyzed using the Mosaic plugin (FIJI) as described previously [19]. The number of doublecortin (DCX)-immunoreactive cells in the subventricular zone (SVZ) was determined on six randomly selected 40 μm sections of each animal. Free-floating sections were treated with 0.6% H2O2 in Tris-buffered saline (TBS; 0.15 m NaCl, 0.1 m Tris–HCl, pH 7.5) for 30 min. Following washes in TBS, sections were blocked with a solution containing TBS, 0.1% Triton-X100 and 3% normal donkey serum solution for 30 min. The latter and goat anti-DCX C-18 (1:500, Santa Cruz Biotechnology, Santa Cruz, USA) primary antibody was applied overnight at 4°C. DCX was visualized by a fluorochrome-conjugated species-specific secondary antibody (Alexa-Fluor 488; Abcam, Cambridge, UK). Sections were mounted on Superfrost Plus slides (Menzel). All DCX-positive cells in the SVZ were manually counted using Eclipse 80i microscope (Nikon).

### Statistics

All analyses except for the LFB analysis were carried out by investigators blinded to the group allocation. Time series of systolic BP and MWM escape latency were analyzed by repeated measures (RM) two-way ANOVA. Microglia count and distribution was analyzed by one and two way ANOVA, respectively. If required, ANOVAs were followed by Bonferroni’s post-hoc tests. The remaining two group comparisons were analyzed by t-tests. A p-value of less than 0.05 was considered statistically significant. All data were displayed as mean ± standard deviation (SD). Data analysis was performed by Graph Pad Prism (version 5.03).

## Results

### Arterial hypertension in SHR is accompanied by cognitive deficits

**Figure 1:**
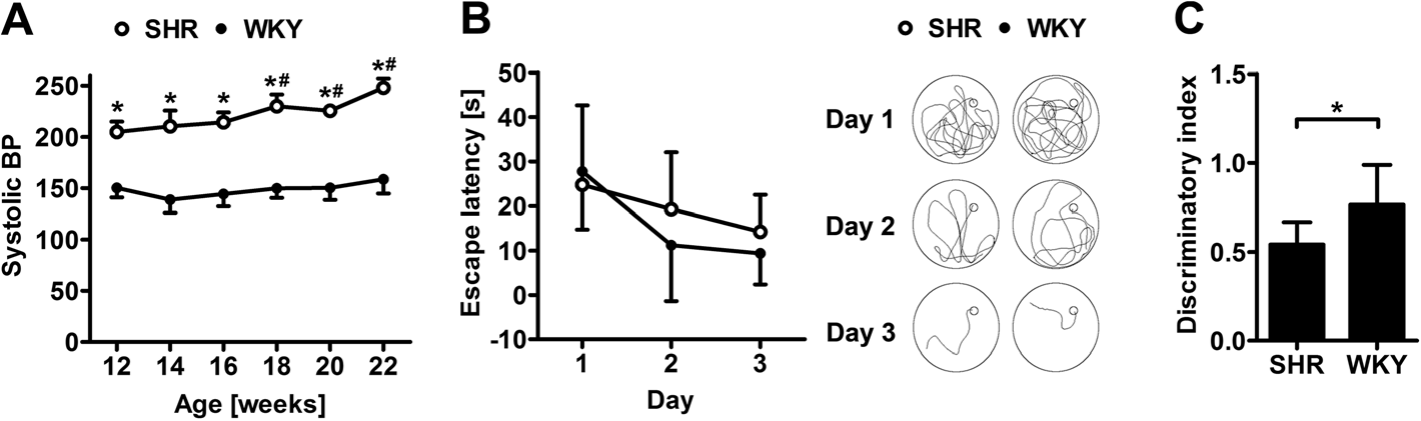
**Spontaneous development of memory deficits in hypertensive rats.** Systolic blood pressure (BP) was significantly higher in SHR at 12 weeks of age and gradually increased thereafter **(A)**. Escape latencies in the Morris water maze (MWM) did not differ between both strains **(B)**. Representative swims paths of SHR and WKY illustrate comparable learning effects over time **(B)**. By contrast, SHR exhibited a significantly impaired discrimination capability in the novel object recognition test **(C)**. Data are mean ± SD. *p < 0.05 versus WKY and ^#^p < 0.05 versus week 12 by two-way RM ANOVA **(A-B)** or t-test **(C)** for n = 9-10 animals/group.

Repeated measurements of the systolic BP in non-anesthetized SHR and age-matched WKY using the tail-cuff method revealed a significant impact of the strain (F(1,18) = 870.71 ; p < 0.001) and the time point of investigation (F(1,18) = 16.04; p < 0.001). SHR exhibited a significantly elevated systolic BP from the age of 12 weeks onwards that further increased at week 18. By contrast, WKY did not show any variation of the systolic BP during the observation period (Figure 1A).

Since cognitive impairment is a common feature of human cSVD, we assessed memory function of both strains by two common tests. In the MWM, the time of analysis (F(1,12) = 6.288; p = 0.006), but not the strain (F(1,12) = 0.548; p = 0.473) significantly influenced escape latencies indicating that hippocampal integrity was mainly preserved in SHR (Figure 1B). By contrast, SHR showed a reduced capability to discriminate novel from previously encountered objects in the NORT suggestive of an impaired non-spatial working memory (Figure 1C).

### SHR exhibit profoundly altered hematological parameters

Basic hematological parameters (Table 1) revealed significantly elevated RBC counts in SHR, but this was not correlated with changes of the Hb or the HCT. The average amount of hemoglobin per RBC (MCH) was decreased and the variation of RBC size (RDW) was increased in SHR. We found almost twice as much platelets in the peripheral blood of SHR, whereas the MPV was significantly smaller when compared to WKY (Table 1). Flow cytometric analyses of blood samples revealed similar leukocyte counts, but considerable differences in some subpopulations. SHR exhibited significantly less T cells, but higher numbers of granulocytes (Table 1). While B and NK cell counts were comparable between SHR and WKY, a trend towards increased monocyte counts in SHR was observed (p = 0.07 by t-test; Table 1).

**Table 1:** **Complete blood count of SHR and WKY**

		**SHR M ± SD**	**WKY M ± SD**	**P**
RBC	10E6/μl	4,71 ± 0,37	4,07 ± 0,12	0,046
Hb	g/dl	8,25 ± 0,41	7,75 ± 0,15	0,118
HCT	%	20,35 ± 1,47	18,27 ± 0,55	0,082
MCH	pg	17,53 ± 0,54	19,13 ± 0,28	0,010
MCHC	g/dl	40,55 ± 1,04	42,55 ± 0,71	0,052
RDW	%	12,78 ± 0,15	12,52 ± 0,06	0,047
PLT	10E5/μl	4,37 ± 0,55	2,86 ± 0,23	0,012
MPV	μm^3^	6,38 ± 0,08	7,03 ± 0,10	0,001
Leukocytes	10E6/mL	8,07 ± 0,85	8,43 ± 0,97	0,649
T cells	10E6/mL	1,48 ± 0,40	3,03 ± 0,29	0,006
B cells	10E6/mL	2,07 ± 0,43	2,41 ± 0,25	0,308
Monocytes	10E6/mL	1,86 ± 0,30	1,13 ± 0,42	0,071
Granulocytes	10E6/mL	1,03 ± 0,14	0,49 ± 0,15	0,011
NK cells	10E6/mL	0,51 ± 0,17	0,63 ± 0,13	0,398

RBC, red blood cell count; Hb, hemoglobin; HCT, hematocrit; MCH, mean corpuscular hemoglobin; MCHC, mean corpuscular hemoglobin concentration; RDW, red blood cell distribution width; PLT, platelet count; MPV, mean platelet volume, p, p-value. Data are mean ± standard deviation, analyzed by t-test.

### Changes of the brain macro- and microstructure in SHR

**Figure 2:**
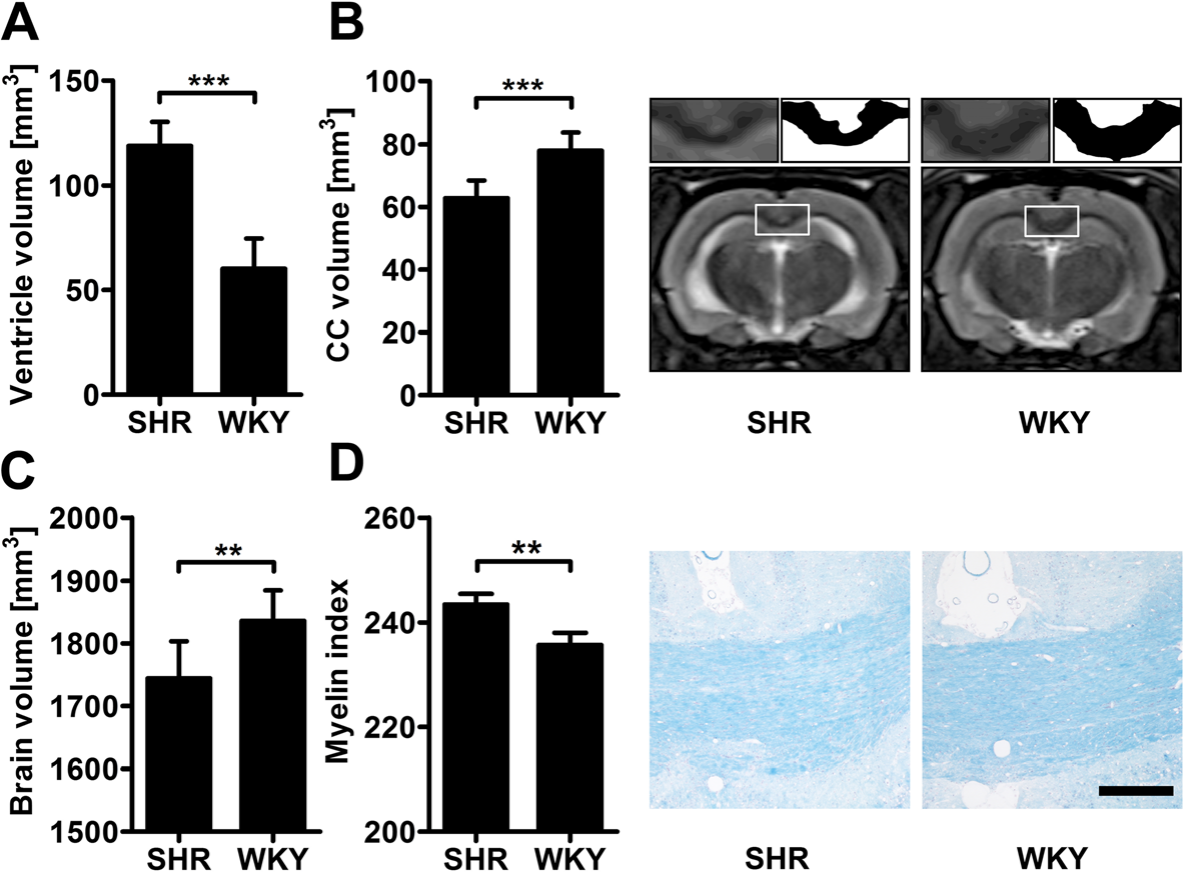
**Structural brain differences between SH (spontaneously hypertensive) and WKY (Wistar Kyoto) rats.** On T2-weighted magnetic resonance imaging (MRI) ventricles were significantly enlarged in SHR **(A)**, whereas corpus callosum (CC) **(B)** and total brain volumes **(C)** were significantly reduced. Representative T2-weighted coronal slices display large hyperintense lateral ventricles in SHR, which are barely visible in WKY. The magnification highlights narrowing of the hypointense CC in SHR. Post-mortal quantification of myelin by Luxol Fast Blue stain revealed a significantly decreased optical density (i.e. an increased myelin index) in the CC of SHR **(D)**. Data are mean ± SD. **p < 0.01 and ***p < 0.001 by t-test for n = 10 (A-C) or n = 4 animals/group **(D)**. Scale bar: 250 μm.

In vivo T2 weighted brain MRI revealed a significant enlargement of the ventricular system in SHR compared to WKY (Figure 2A). Furthermore, SHR exhibited smaller corpora callosa and brain volumes (Figure 2B and C). The latter finding was corroborated by the assessment of brain weights post mortem (SHR: 1952 ± 299 mg versus WKY: 2282 ± 141 mg; p = 0.011 by t-test). LFB staining disclosed that the decline in CC volume among SHR was accompanied by a significant myelin loss (Figure 2D).

**Figure 3:**
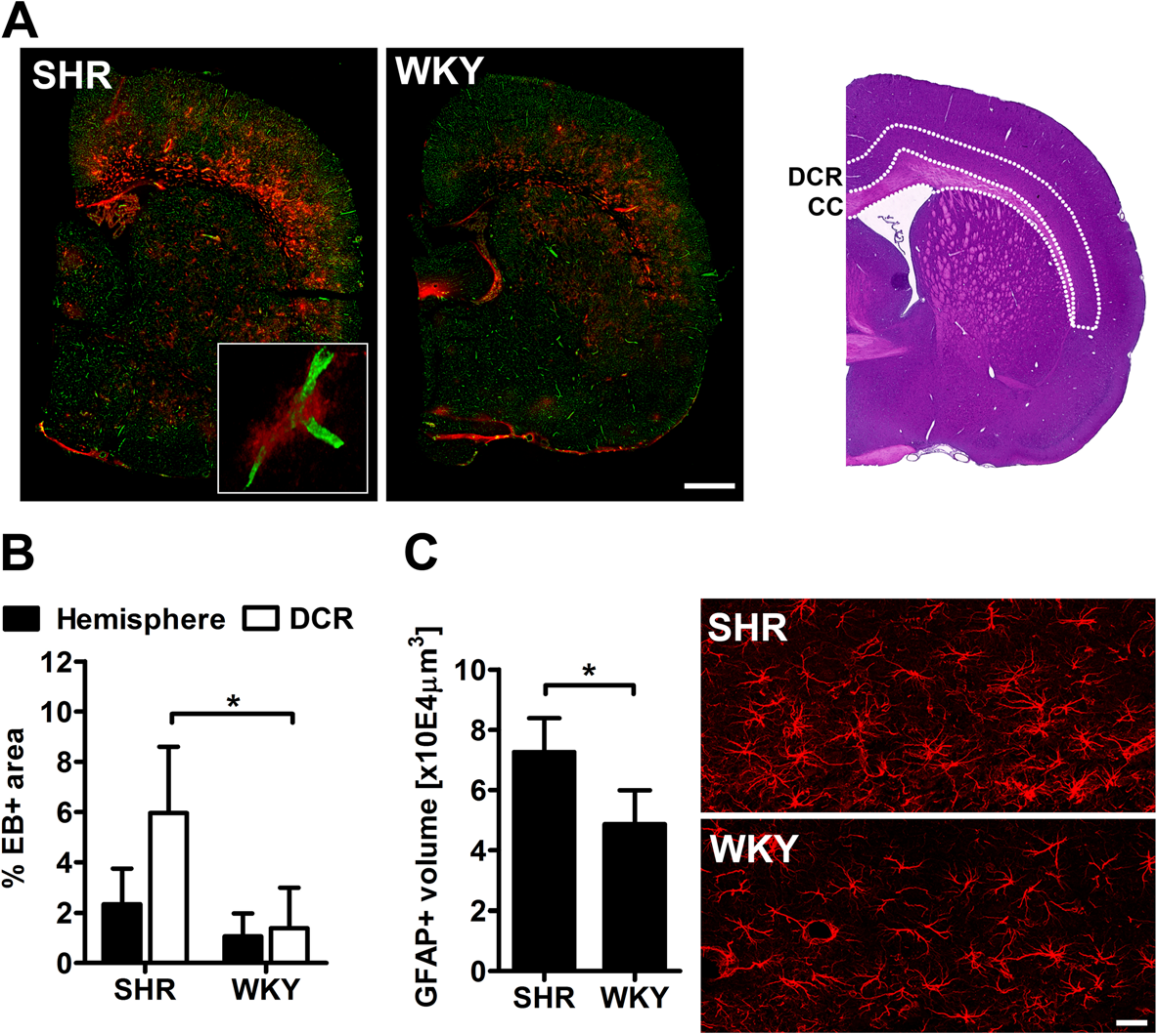
**Circumscribed blood brain barrier (BBB) leakage and astroglial activation in the deep cortical region (DCR) of SHR. (A)** shows representative micrographs of whole hemispheres stained with Evans blue (EB, red) and FITC-lectin (green). Note the increased EB extravasation in the DCR adjacent to the corpus callosum (CC) in SHR. Quantification of BBB permeability by the EB method revealed no strain-dependent differences when comparing entire hemispheres **(B)**. However, the percent EB positive area was significantly increased in the DCR of SHR **(B)**. Astrogliosis was determined by immunofluorescence staining of GFAP (red) **(C)**. Compared to WKY, the GFAP-positive volume in the DCR of SHR was significantly enhanced. Data are mean ± SD. *p < 0.05 by t-test for n = 3 **(B)** or n = 4 animals/group **(C)**, scale bar: 1 mm **(A)**, 20 μm **(C)**.

**Figure 4:**
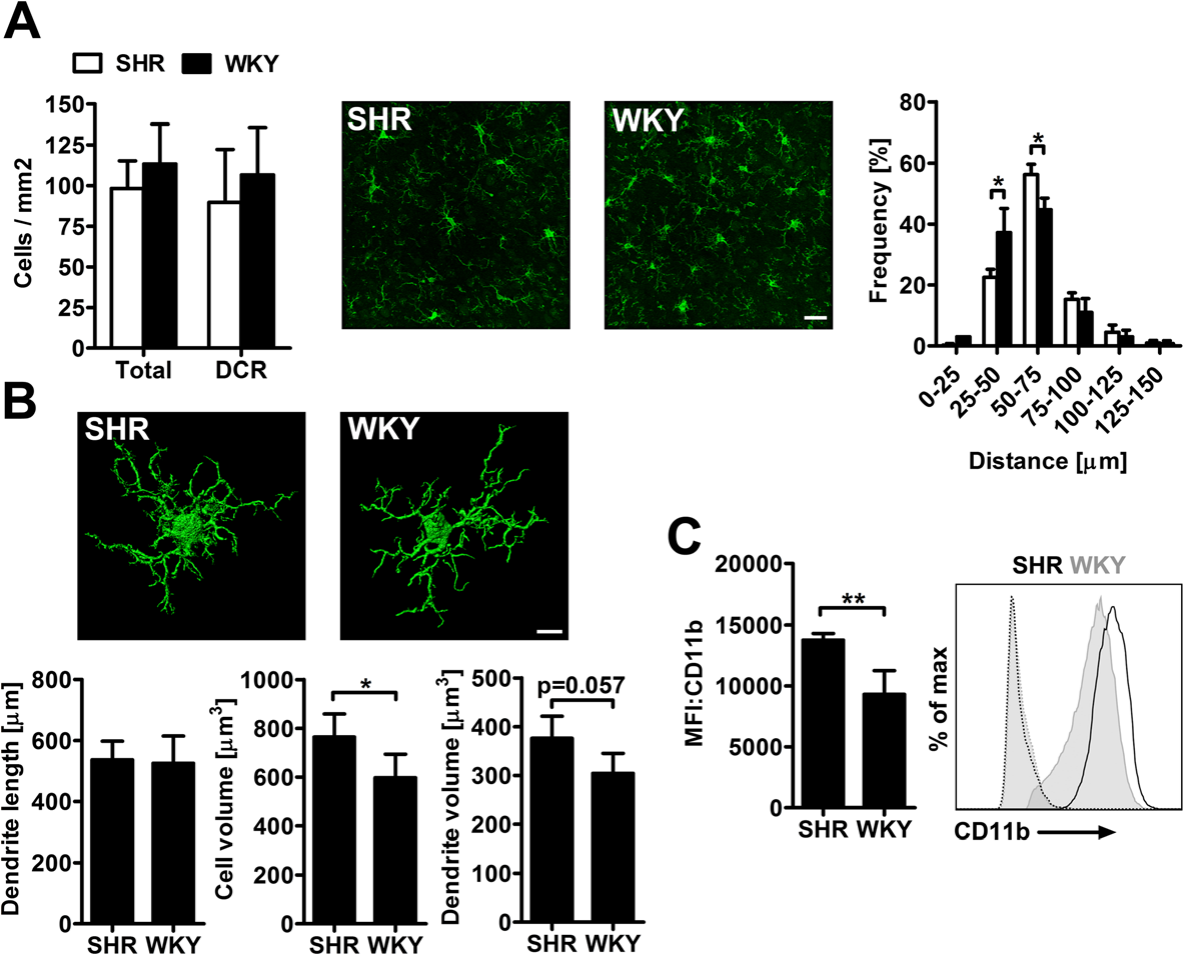
**Microglial rarefication and activation in the brain of SHR.** Total amounts of Iba1-positive microglia (green) in the total brain and deep cortical region (DCR) did not differ strain-dependently **(A)**. However, distances between adjacent cells were significantly greater in SHR compared to WKY **(A)**. Single cell morphological analysis of microglia within the DCR showed similar dendrite lengths, but increased cell volumes in SHR **(B)**. **(C)** Mean fluorescence intensities (MFI) of microglial CD11b expression were significantly higher in SHR. The representative histogram illustrates the shift towards increased CD11b expression in SHR. Data are mean ± SD. *p < 0.05, **p < 0.01 by one or two-way ANOVA **(A)** or t-test **(B and C)** for n = 4 animals/group, scale bar: 30 μm **(A)**, 8 μm **(B)**.

Analysis of BBB integrity by the EB method revealed comparable amounts of dye extravasation per hemisphere, but an increased leakage in the CC and the adjacent parenchyma hereafter referred to as deep cortical region (DCR; Figure 3A). Compared to WKY, EB extravasation was significantly enhanced in the DCR of SHR indicating a circumscribed BBB leakage within that region (Figure 3B). To further explore astrocyte activation within the DCR, we calculated three-dimensional reconstructions of astrocytes visualized by GFAP staining and found that astrogliosis was significantly induced in SHR (Figure 3C). Quantification of Iba1+ brain microglia showed comparable cell counts in the DCR and the total brain of both strains. However, a nearest-neighbor analysis revealed that microglia is less contiguous in SHR compared to WKY (Figure 4A). The latter finding was evident in both, the DCR and the remaining brain tissue (data not shown). Single cell morphological analysis of microglia within the DCR indicated comparable dendrite lengths, but increased cellular volumes (Figure 4B) as indicative of microglial hypertrophy. Microglial activation was further confirmed by flow cytometry showing a significantly increased CD11b expression [20] of brain microglia in SHR (Figure 4C).

### CNS leukocyte counts and distribution are different in SHR and WKY

**Figure 5:**
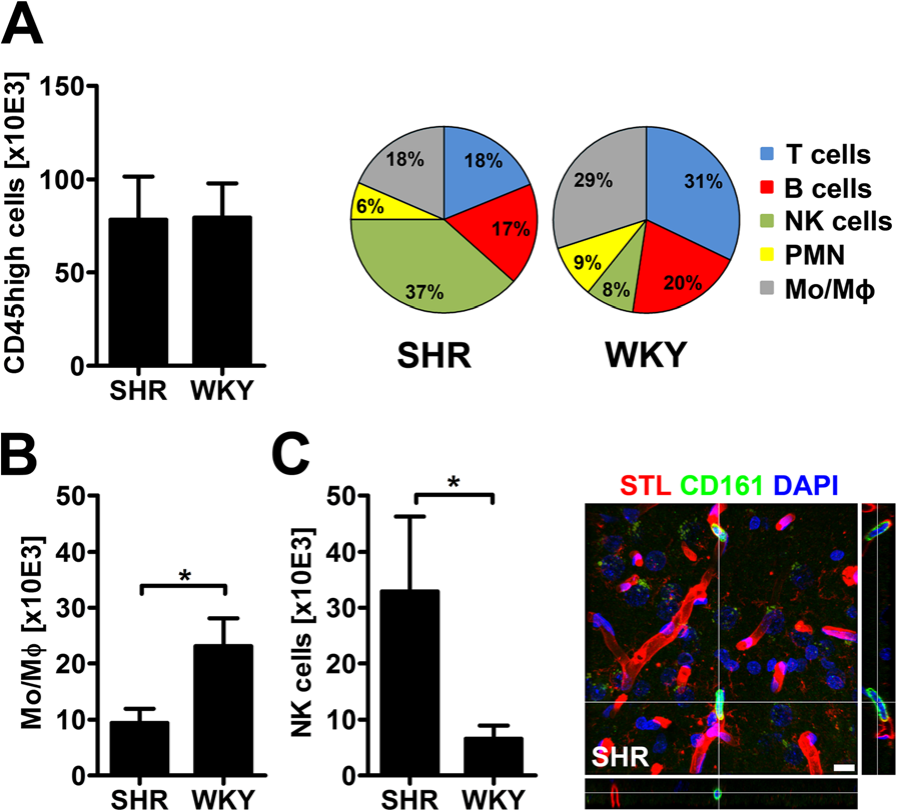
**Brain leukocyte subpopulations differ between SHR and WKY.** Brains of SHR and WKY contained comparable numbers of CD45 high leukocytes while the composition of subpopulations was strongly different **(A)**. NK cells constituted the predominant immune cell type in SHR. By contrast, the proportion of T cells and monocytes/macrophages (Mo/Mɸ) was higher in WKY. Absolute counts of Mo/Mɸ were decreased **(B)** whereas NK cells were significantly increased in brains of SHR **(C)**. Immunofluorescence analysis revealed that CD161 positive NK cells (green) were exclusively located in the brain microvasculature (visualized by solanum tuberosum lectin, STL; red) of SHR. Data are mean ± SD. *p < 0.05 by t-test for n = 3 animals/group, scale bar: 10 μm. PMN, polymorphonuclear granulocytes.

Flow cytometric analysis revealed comparable CD45high leukocyte counts in brain lysates of SHR and WKY (Figure 5A). However, the composition of subpopulations was clearly different. While the proportion of T cells and CD11b + mononuclear cells (monocytes and macrophages) was higher in WKY, we found that NK cells constituted the predominant immune cell population in brains of SHR (Figure 5A-C). Immunohistochemical studies indicated that NK cells in brains of SHR were exclusively located within the microvasculature and exhibited an elongated rod-like form (Figure 5C).

**Figure 6:**
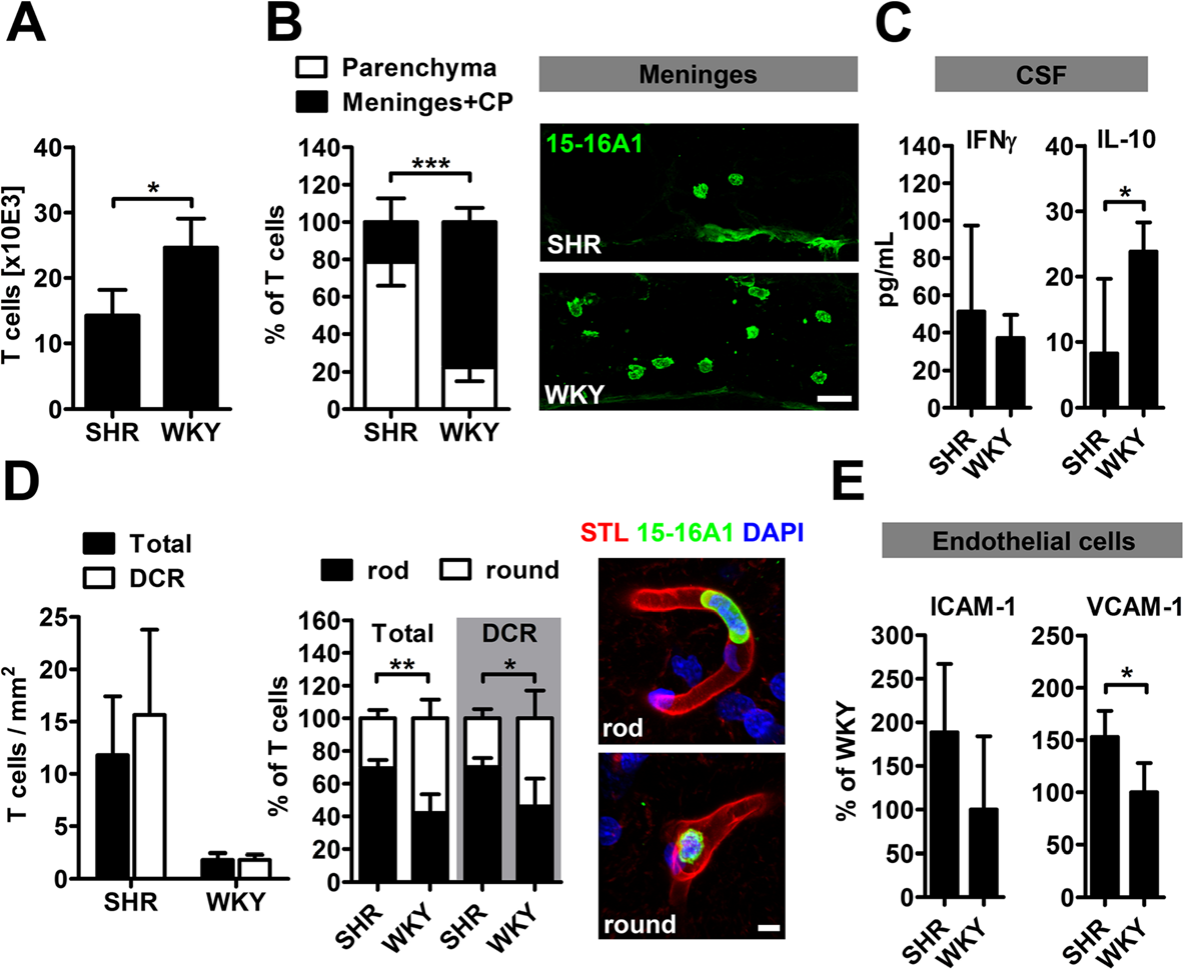
**T cell compartmentalization and morphology is different in SHR.** Absolute T cell counts were significantly lower in brains of SHR **(A)**. Histological analysis of T cell distribution in both strains revealed that only 20% of total T cells in SHR resided in the meninges and the choroid plexus (CP), while the remaining cells were located in parenchymal microvessels **(B)**. This distribution was reversed in WKY. **(B)** shows representative sections of the meninges in SHR and WKY. T cells were identified by the pan T cell marker 15-16A1 (green). Distinct T cell compartmentalization was accompanied by a decrease of IL-10, but similar levels of IFNγ in the cerebrospinal fluid (CSF) of SHR **(C)**. Numbers of intravascular T cells neither differed in the total brain nor in the deep cortical region (DCR) **(D)**. In SHR, two-thirds of all T cells were rod-shaped while the rod-to-round ratio was almost 1:1 in WKY **(D)**. Representative images of rod and round shaped T cells (green) in brain microvessels (visualized by solanum tuberosum lectin, STL; red). mRNA expression of vascular adhesion molecule 1 (VCAM-1) was significantly increased in brain endothelial cells of SHR **(E)**. Data are mean ± SD. *p < 0.05, **p < 0.01, ***p < 0.001 by t-test for n = 3 **(A)**, n = 4 **(B, C and D)** or n = 5 **(E)** animals/group, scale bar: **(B)** 20 μm; **(D)** 5 μm.

Absolute T cell counts were higher in WKY brains (Figure 6A), but further histological analyses revealed remarkable differences in the distribution of T cells among both strains. In normotensive WKY, the majority of T cells was found in the choroid plexus (CP) and the meninges, whereas in SHR, most of the T cells resided in the parenchymal microvasculature (Figure 6B). The diverse compartmentalization of T cells was accompanied by lower levels of the anti-inflammatory Th2 cytokine IL-10, but similar concentrations of the Th1 cytokine IFNγ in the CSF of SHR (Figure 6C). Next, we investigated the distribution and morphology of T cells in the brain microvasculature and found equal cell counts in the DCR and the total brain (Figure 6D). In SHR, two-thirds of all T cells were rod-shaped, indicating an advanced stage of the leukocyte transmigration sequence [21]. By contrast, the rod-to-round ratio was almost 1:1 in WKY. This ratio was similar in the DCR and the remaining brain (Figure 6D). T cell transmigration across the brain endothelium was only sporadically observed (Additional file 2: Figure S2). Because T cell adhesion and trafficking is primarily controlled by endothelial adhesion molecules, we analyzed the mRNA expression of ICAM, VCAM and P-selectin in brain endothelial cells after laser microdissection. Interestingly, we found distinct expression patterns with equal expression of ICAM-1, but upregulation of VCAM-1 (Figure 6E) and reduced expression of P-Selectin in hypertensive animals (SHR 8.4 ± 18.8% versus WKY 100 ± 77.9%, p = 0.036 by t-test).

### Evidence for concomitant tissue repair and endothelial dysfunction in brains of SHR

**Figure 7:**
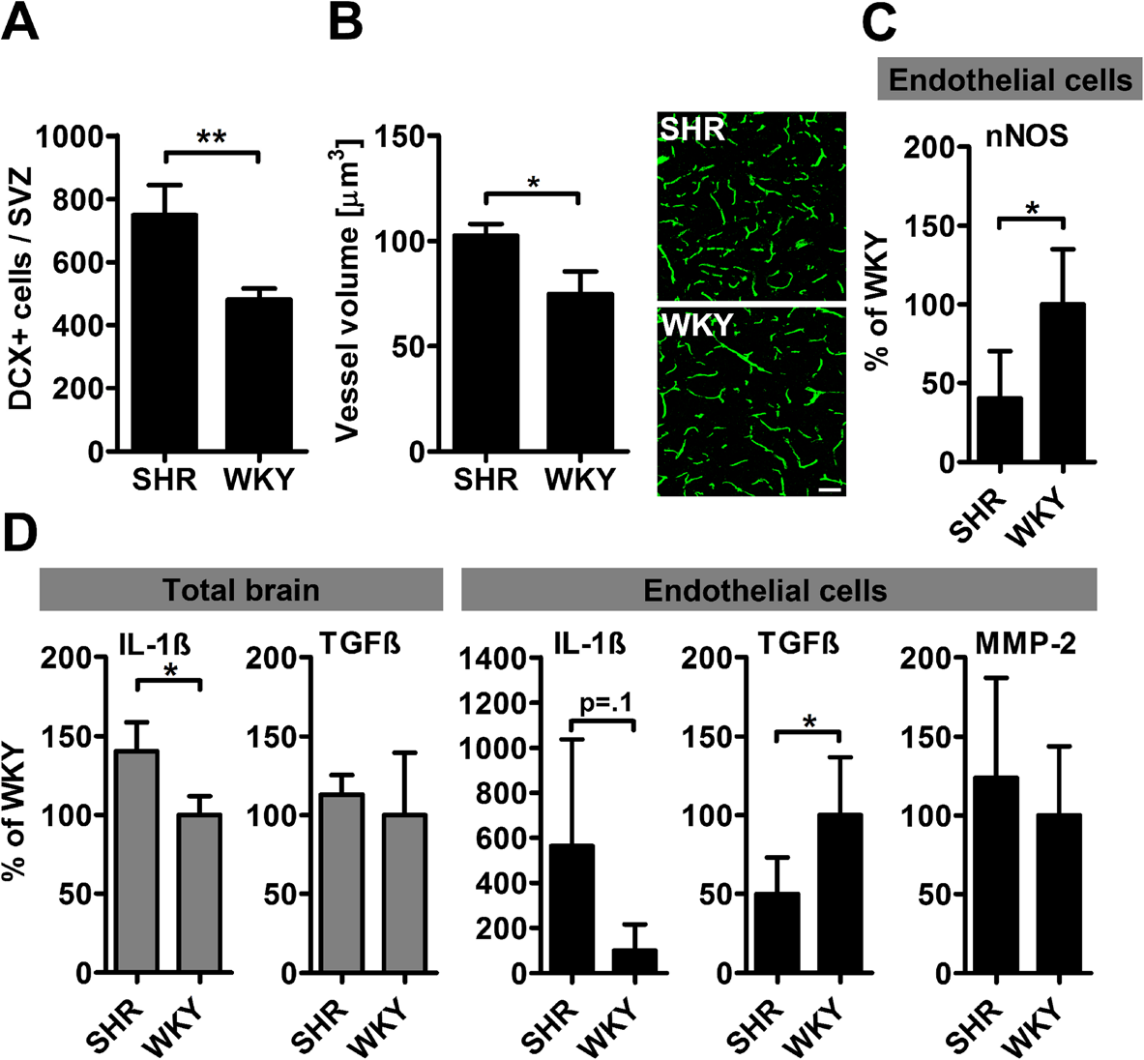
**Repair and inflammation in brains of SHR.** Neurogenesis as measured by the number of doublecortin (DCX)-expressing cells in in the subventricular zone (SVZ) was increased in SHR **(A)**. Similarly, brains of SHR exhibited larger vessel volumes, assessed by three-dimensional reconstruction of FITC-lectin stained endothelium **(B)**. Neuronal nitric oxide synthase (nNOS) was significantly down-regulated in endothelial cells of SHR **(C)**. IL-1β expression was significantly increased in SHR whole brain lysates, whereas the expression of TGFβ was comparable. In SHR endothelial cells, there was a non-significant increase of IL-1β expression and a significantly decreased expression of TGFβ. Endothelial matrix metalloproteinase (MMP)-2 expression did not differ between SHR and WKY **(D)**. Data are mean ± SD. *p < 0.05 and **p < 0.01 by t-test for n = 3-5 animals/group. Scale bar: 50 μm.

We finally analyzed processes counter-regulating inflammation and allowing brain repair. The amount of DCX-positive neural progenitors within the SVZ was significantly increased in SHR (Figure 7A). Moreover, the microvasculature of hypertensive animals in the DCR differed markedly from that of WKY. Besides an increase in the wall-to-lumen ratio (SHR: 8.56 ± 1.30 versus WKY: 5.50 ± 1.04; p = 0.03 by t-test), we observed an augmentation of vessel volumes in SHR (Figure 7B). Neuronal nitric oxide synthase (nNOS), an important source of endothelial nitric oxide [22] was significantly down regulated in brain endothelium of SHR. Gene expression of IL-1β was significantly increased in whole brain lysates of SHR (p < 0.05; Figure 7D), but not in endothelial cells (p = 0.1). Immunohistochemistry identified astrocytes as major source of IL-1β (Additional file 3: Figure S3). While mRNA expression of TGFβ was comparable in the entire brain, it was significantly decreased in endothelial cells of SHR. Endothelial matrix metalloproteinase (MMP)-2 expression did not differ between SHR and WKY (Figure 7D).

## Discussion

We found first experimental evidence that SHR spontaneously develop circumscribed BBB leakage, white matter loss and cerebrovascular inflammation, accompanied by memory deficits already in their first third of life. SHR thus share many similarities with cSVD patients in which a strong correlation between elevated blood pressure, brain atrophy and cognitive decline was shown [23,24].

Microvascular brain damage is commonly considered a consequence of large artery stiffness and increased pulse pressure. The latter accounts for pulsatile stress in the microcirculation of high flow organs such as the brain and induces remodeling of small vessel walls [25,26]. Untreated hypertension accelerates this process and thus perpetuates a vicious cycle of progressive arterial aging and rising blood pressure [27]. Besides invasive hemodynamic monitoring, altered erythrocyte parameters can be measured as surrogate markers for target organ damage in hypertension. In line with clinical observations [28,29], we found higher red blood cell counts and an increased RDW in SHR, corroborating the assumption that similar biophysical conditions determine microvascular damage in SHR and hypertensive patients.

Wall hypertrophy and luminal diameter reduction of small penetrating arteries are findings considered pathognomonic for hypertensive cSVD [30]. A common hypothesis is that these microvascular changes result in a state of chronic hypoperfusion leading to oligodendrocyte death and consecutive degeneration of myelinated fibers [31]. Indeed, we observed demyelination and volume loss of the CC in SHR, conforming to white matter damage as another important hallmark of human cSVD [30]. Additionally, SHR exhibited global brain atrophy and ventricular enlargement on T2-weighted MRI. However, our MRI investigations neither revealed lacunar infarcts nor white matter hyperintensities as key imaging features of cSVD up to 35 weeks of age. One possible explanation is that subtle white matter damage, as unambiguously present on post mortem brain tissue of SHR, escaped detection in our study because T2-weighted MRI is relatively insensitive towards changes of the brain microstructure. A recent clinical study supports this idea by providing convincing evidence that changes of the white matter microstructure quantifiable by diffusion tensor imaging temporally precede the development of T2-visible white matter lesions [32].

A large cross-sectional imaging study revealed that increased systolic BP affects white matter microstructure and grey matter volume already in young adults. Brain injury preferentially occurred in the anterior CC and other frontal white matter tracts being associated with cognitive decline later in life [11]. In our study, middle-aged SHR showed a reduced discrimination capability in the NORT. This test evaluates non-spatial working memory primarily related to frontal subcortical circuits [33] which are commonly affected in patients with hereditary cSVD [34], but are also particularly susceptible to hypertensive brain damage [11].

By contrast, performance in the MWM was similar to age-matched normotensive controls, suggesting that the integrity of the hippocampus is largely preserved in SHR up to 35 weeks of age. This was surprising since several clinical trials found evidence for an association between elevated blood pressure, white matter lesions and reduced hippocampal volume [11,35]. Moreover, a prior histological study observed time-dependent loss of cornu ammonis 1 pyramidal neurons in SHR [36]. We did not assess hippocampal volumes in this study, however, even if cell loss was present it did not translate into abnormalities in the MWM. One could speculate that destruction of hippocampal neurons in middle-aged SHR with few months of hypertension might not have passed a threshold beyond which clinical decompensation occurs. However, our study is hampered by assessment of cognitive performance at only one time point, thus future studies investigating hippocampal integrity and cognitive function in a longitudinal manner are needed to clarify this issue. They might also help to reliably establish the relationship between cognitive decline and blood pressure levels.

Cognitive testing in SHR is additionally complicated by the fact that they exhibit certain behavioral features of attention deficit hyperactivity disorders (ADHD) [37]. These behavioral abnormalities are present early in life and are commonly ascribed to dopaminergic hypofunction in SHR [38]. Executive dysfunction and attention deficits are symptoms partly shared by ADHD [39] and cSVD [40,41], however, motor hyperactivity and impulsiveness do not belong to the neuropsychological profile of patients with cSVD. Thus, concomitant neurotransmitter dysregulations in SHR might limit its usefulness as an experimental model for cSVD.

The idea of an anatomically predisposed region in early stages of hypertensive cSVD [11,42] was further supported by our finding of a circumscribed BBB leakage in SHR. We found a significant increase of albumin extravasation in the DCR adjacent to the CC while the total BBB function was comparable between SHR and age-matched normotensive controls. The structure of arterioles entering deep brain regions from the superficial cortex particularly exposes them to the effects of vascular disease and might relate to this predilection site [4,10]. The DCR was also characterized by a significant astrogliosis and activation of microglia. This finding suits well with recent work that stressed the close association of local BBB disruption and inflammation with oligodendrocyte death following unilateral carotid artery occlusion in SHR-SP [31]. In our study, activated astrocytes were the primary source of the central pro-inflammatory cytokine IL-1β which was significantly increased in brain parenchyma of SHR. Similar findings were previously obtained in rats with angiotensin II induced hypertension [43]. In the brain, IL-1β directly influences BBB permeability [44], antagonizes endothelial anti-inflammatory TGFβ signaling [45] and increases arterial BP [43]. Circumscribed neuroinflammation and IL-1β signaling hence seem to play an important role in the pathophysiological cascade of hypertensive brain damage and the further maintenance of hypertension.

Beyond the activation of brain-resident cells, we found substantial differences in the amount and composition of blood-borne leukocytes between SHR and normotensive controls. In WKY, almost twice as much T cells were detectable in brain lysates, but immunohistochemistry revealed that they were mostly localized within the meninges and CP. By contrast, in SHR the vast majority of T cells populated the microvessels within the brain parenchyma, and could be further distinguished into round and rod shaped cells. The latter morphological discrimination may represent two stages of the leukocyte margination sequence [21], and firm adhesion to the vessel wall may have hampered their removal during the perfusion. The antithetic distribution of T cells in brains of SHR and WKY could be well explained by different expression patterns of endothelial adhesion molecules. We found an upregulation of VCAM-1 in brain endothelial cells of SHR, likely as consequence of an activated renin angiotensin system during arterial hypertension [46]. This finding is supported by clinical evidence for increased levels of soluble VCAM-1 in cSVD patients [47]. Increased expression of adhesion molecules, and possibly also a slowed vascular transit time of leukocytes due to pseudopod formation [48] are conclusive explanations for the ample occurrence of intravascular T cells in SHR brains. On the other hand, T cells adhering to the luminal side of cerebral microvessels might be part of the systemic adaptive immune response against vascular neoantigens during hypertension. T cells directly promote endothelial dysfunction, but may also compromise brain perfusion by microvascular plugging and thrombosis [49,50]. Importantly, only the minority of T cells crossed the brain endothelium in our study. Moreover, there was no specific accumulation of T cells within the inflamed DCR. Collectively, these findings argue against a CNS-directed autoreactive T cell response in the early stages of hypertensive neuroinflammation.

Another interesting finding was the considerable decrease of T cells in the meningeal space and the CP of SHR. This observation was accompanied by lower concentrations of the anti-inflammatory cytokine IL-10 in the CSF. Meningeal T cells have significant impact on learning behavior, memory function and mood stabilization [51,52]. It is therefore tempting to speculate that cognitive deficits in hypertensive individuals could be, at least partly, a consequence of a disturbed CNS immune homeostasis. T cell trafficking via the endo- and epithelium of the CP to the CSF is highly dependent on P-selectin [53] and P-selectin polymorphisms have been associated with cognitive dysfunction [54,55]. In contrast to VCAM-1 and ICAM-1, P-selectin was significantly down regulated in the cerebral microvasculature of SHR in our study. This finding is supported by previous studies and might be related to increased levels of corticosteroids in SHR [56]. Further research is required to shed light on the relation between cerebrovascular P-selectin expression and cognitive decline in the context of hypertensive brain damage.

Another interesting result is the high amount of NK cells in brains of SHR. The majority of these cells appeared elongated and was located in the vessel lumina. A recent study described an important cross-talk between monocytes and NK cells during angiotensin II induced vascular inflammation in the aortic wall. IL-12 secreting monocytes attract and activate NK cells which sustain vascular dysfunction by IFNγ production [57]. However, little is known about the role of NK cells in cSVD. While previous studies described that brains of hypertensive rats exhibit significantly more perivascular monocytes/macrophages compared to age-matched controls [58], we found lower numbers of monocytes in SHR whole brain lysates. Moreover, we did not observe myeloid cells that adhered to the vessel walls in SHR, indicating that NK cells and myeloid cells sojourn in different compartments of the neurovascular unit. However, hypertension-related IFNγ production by either NK cells or T cells [59] could possibly explain the low expression of endothelial nNOS in brains of hypertensive animals seen in our study. Downregulation of nNOS in turn increases endothelial susceptibility for inflammatory cytokines and enhances expression of VCAM-1 [22].

Besides their direct participation in endothelial dysfunction, T cells and NK cells might play an important role in cerebral arteriogenesis [60] owing to perfusion deficits and shear stress. In contrast to a recent study [61], we actually observed evidence for arteriogenesis in the DCR. However, cellular and molecular alterations as a result of hypertension-related vascular dysfunction can hardly be discriminated from changes induced by arteriogenesis, since the latter process is also driven by inflammation [62]. Neurogenesis is another counter-regulatory mechanism which might be initiated by white matter injury in cSVD [63]. In fact, a previous study demonstrated enhanced hippocampal neurogenesis in hypertensive rats [64]. We found an increase of DCX-positive neural progenitors in the SVZ of SHR further supporting the idea that hypertensive brain damage is accompanied by repair processes. One might assume that reduced endothelial TGFβ expression due to hypertension-related inflammation as seen in our study alters the neurovascular niche and finally promotes neurogenesis [65]. However, if this is the case and whether these repair mechanisms delay the disease process and possible clinical thresholds of cSVD needs to be elucidated in future studies.

## Conclusion

**Figure 8:**
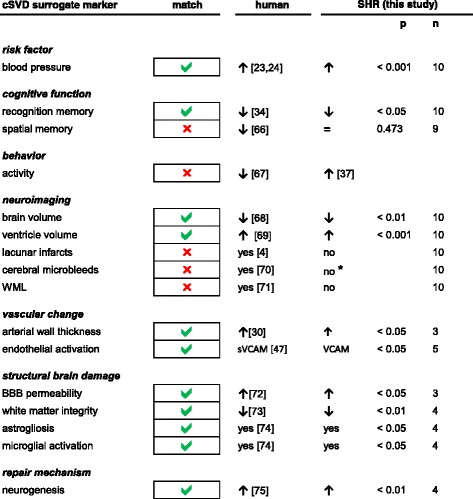
**SHR share key features with patients with hypertensive cerebral small vessel disease (cSVD).** Results of this study include the number of biological replicates (n) and the p-value (p). (s)VCAM, (soluble) vascular adhesion molecule; WML, white matter lesions, BBB, blood brain barrier. * on histological sections.

In conclusion, our study shows that hypertensive rats spontaneously develop brain atrophy and white matter loss as well as BBB leakage, astrogliosis and microglial activation in the deep grey matter. They thus share many neuropathological features with hypertensive brain damage and cSVD in humans. Importantly, these changes of the brain macro- and microstructure were already apparent early in life indicating that SHR might serve as a valid experimental model for early-stage cSVD. Neuroimaging and cognitive testing partially revealed conflicting results, but when considering overall findings of our study, similarities with the human disease clearly outweigh inconsistencies (Figure 8) [66-75]. Transfer of findings from rats to humans remains difficult due to obvious species differences, however, we believe that certain aspects of human cSVD pathophysiology are well reflected in SHR.

We additionally found evidence for endogenous repair mechanisms in SHR which might delay disease manifestation and progression. Furthermore, our results suggest a role of NK and T cells for cerebrovascular inflammation and hypertension-related cognitive decline. Due to the descriptive character of our study, it remains to be proven whether these changes causally contribute to disease development or rather represent an epiphenomenon of the disease process. However, if future research confirms the contribution of the immune system to the initiation and propagation of cSVD, new therapeutic approaches may arise.

## Additional files

Additional file 1: Figure S1. Outline of the experimental design. In vivo investigations (green) included measurement of systolic blood pressure (BP), novel object recognition test (NORT), Morris water maze (MWM) and magnetic resonance imaging (MRI). The cross sign indicates animal sacrifice. Post mortem analyses are indicated in red. WKY, Wistar Kyoto rats; SHR, spontaneously hypertensive rats; CSF, cerebrospinal fluid; FACS, flow cytometry; LMD/PCR, laser microdissection and gene expression analysis. Additional file 2: Figure S2. Representative example for T cell (15-16A, green) transmigration across the brain endothelium (STL, solanum tuberosum lectin, red). Left: orthographic projection; right: superposition of a confocal z-stack). Nuclei are counterstained with DAPI. Scale bar: 5 μm. Additional file 3: Figure S3. Representative example for IL-1β expression in brains of SHR and WKY. IL-1β (green) colocalizes well with astrocytes (GFAP, red), but not with microglia and endothelium (STL, solanum tuberosum lectin, magenta). Nuclei are counterstained with DAPI. Merged image: superposition of a confocal z-stack; IL-1β, STL and GFAP: rendered objects of the same stack. Scale bar: 10 μm.
