# Supplementary material for: Spontaneous white matter damage, cognitive decline and neuroinflammation in middle-aged hypertensive rats: an animal model of early-stage cerebral small vessel disease
Source: Acta Neuropathol Commun. 2014 Dec 18;2:169. doi: 10.1186/s40478-014-0169-8 (PMC4279586; doi:10.1186/s40478-014-0169-8)
Supplement: Additional file 1: Figure S1. — Outline of the experimental design. In vivo investigations (green) included measurement of systolic blood pressure (BP), novel object recognition test (NORT), Morris water maze (MWM) and magnetic resonance imaging (MRI). The cross sign indicates animal sacrifice. Post mortem analyses are indicated in red. WKY, Wistar Kyoto rats; SHR, spontaneously hypertensive rats; CSF, cerebrospinal fluid; FACS, flow cytometry; LMD/PCR, laser microdissection and gene expression analysis. [file 40478_2014_169_MOESM1_ESM.doc]

Additional file 1: Figure S1


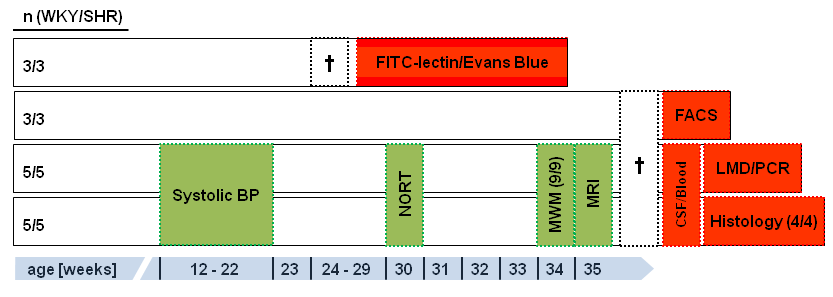


Supplementary Figure 1. Outline of the experimental design.
In vivo investigations (green) included measurement of systolic blood pressure (BP), novel object recognition test (NORT), Morris water maze (MWM) and magnetic resonance imaging (MRI). The cross sign indicates animal sacrifice. Post mortem analyses are indicated in red. WKY, Wistar Kyoto rats; SHR, spontaneously hypertensive rats; CSF, cerebrospinal fluid; FACS, flow cytometry; LMD/PCR, laser microdissection and gene expression analysis.
