# Supplementary material for: Spontaneous white matter damage, cognitive decline and neuroinflammation in middle-aged hypertensive rats: an animal model of early-stage cerebral small vessel disease
Source: Acta Neuropathol Commun. 2014 Dec 18;2:169. doi: 10.1186/s40478-014-0169-8 (PMC4279586; doi:10.1186/s40478-014-0169-8)
Supplement: Additional file 2: Figure S2. — Representative example for T cell (15-16A, green) transmigration across the brain endothelium (STL, solanum tuberosum lectin, red). Left: orthographic projection; right: superposition of a confocal z-stack). Nuclei are counterstained with DAPI. Scale bar: 5 μm. [file 40478_2014_169_MOESM2_ESM.doc]

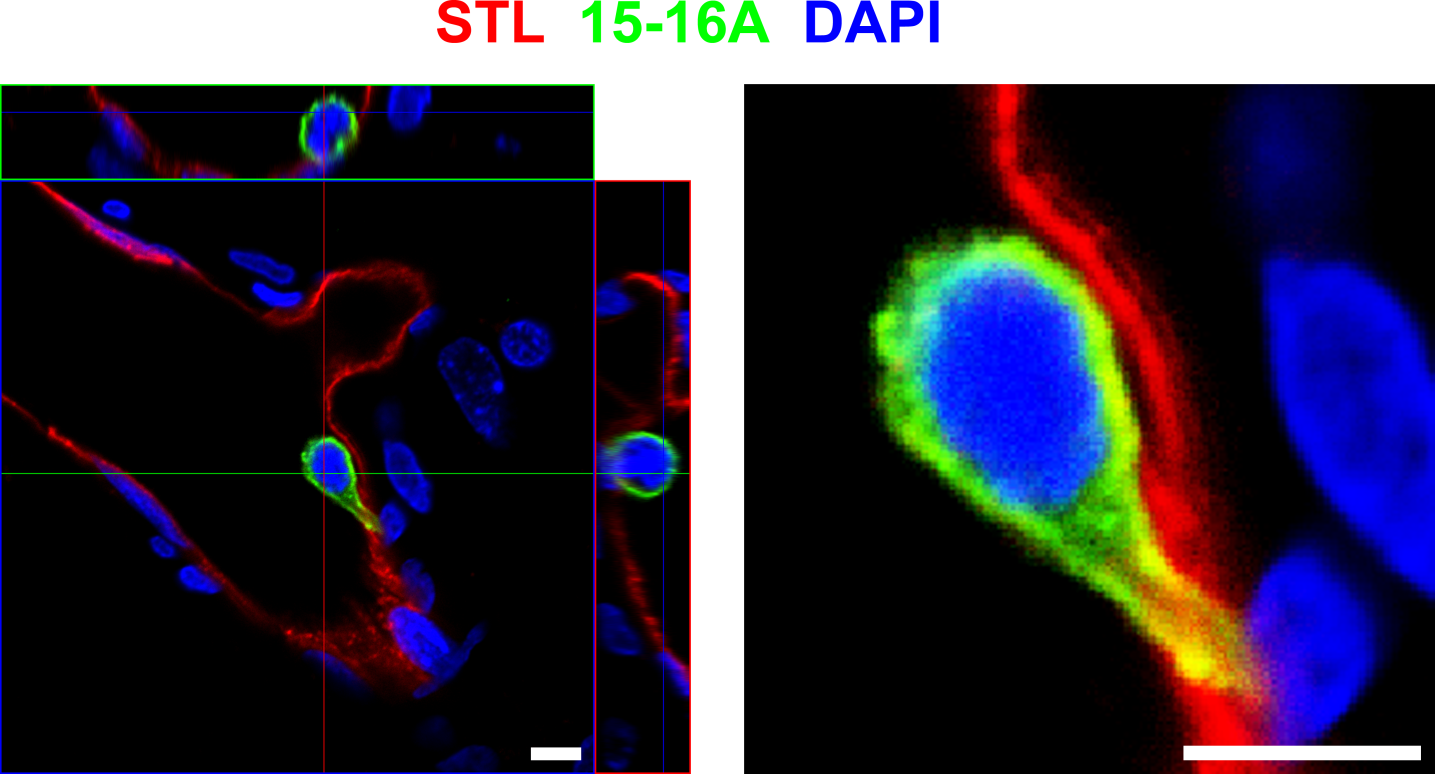


Additional file 2: Figure S2. Representative example for T cell (15-16A, green) transmigration across the brain endothelium (STL, solanum tuberosum lectin, red). Left: orthographic projection; right: superposition of a confocal z-stack). Nuclei are counterstained with DAPI. Scale bar: 5 µm.
