# Supplementary material for: Spontaneous white matter damage, cognitive decline and neuroinflammation in middle-aged hypertensive rats: an animal model of early-stage cerebral small vessel disease
Source: Acta Neuropathol Commun. 2014 Dec 18;2:169. doi: 10.1186/s40478-014-0169-8 (PMC4279586; doi:10.1186/s40478-014-0169-8)
Supplement: Additional file 3: Figure S3. — Representative example for IL-1β expression in brains of SHR and WKY. IL-1β (green) colocalizes well with astrocytes (GFAP, red), but not with microglia and endothelium (STL, solanum tuberosum lectin, magenta). Nuclei are counterstained with DAPI. Merged image: superposition of a confocal z-stack; IL-1β, STL and GFAP: rendered objects of the same stack. Scale bar: 10 μm. [file 40478_2014_169_MOESM3_ESM.doc]

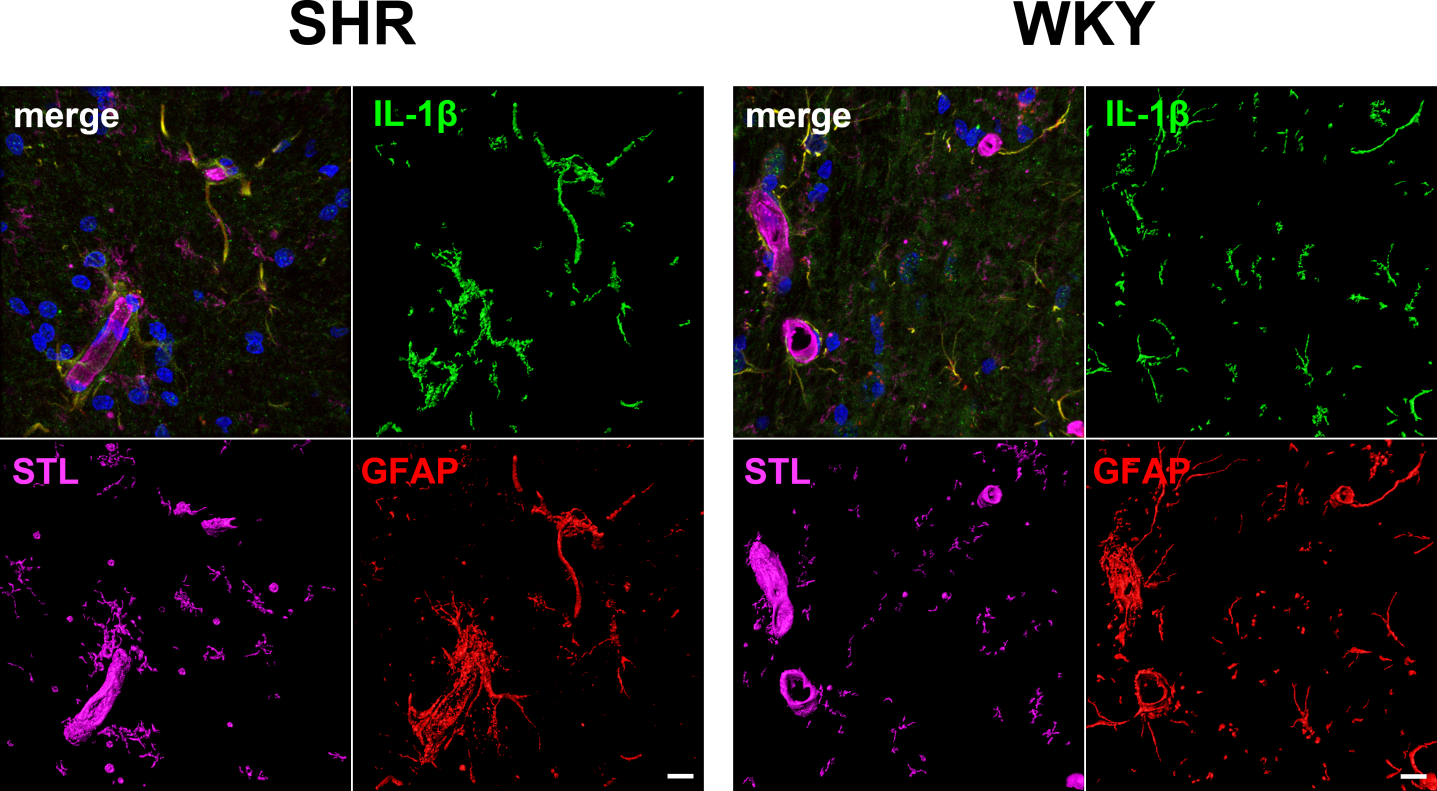


Additional file 3: Figure S3. Representative example for IL-1β expression in brains of SHR and WKY. IL-1β (green) colocalizes well with astrocytes (GFAP, red), but not with microglia and endothelium (STL, solanum tuberosum lectin, magenta). Nuclei are counterstained with DAPI. Merged image: superposition of a confocal z-stack; IL-1β, STL and GFAP: rendered objects of the same stack. Scale bar: 10 µm.
